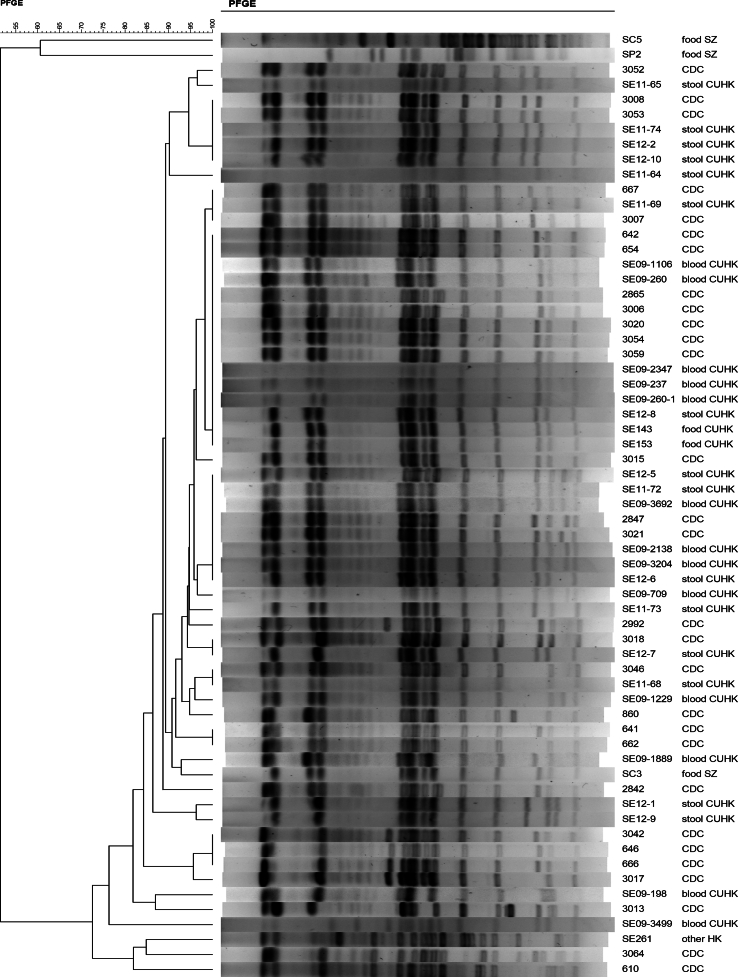# Novel virulence-related genes that contribute to clinical infections of *Salmonella* enteritidis

**DOI:** 10.1016/j.gendis.2024.101236

**Published:** 2024-02-01

**Authors:** Bill Kwan-wai Chan, Ruichao Li, Edward Wai-chi Chan, Kwok-yin Wong, Sheng Chen

**Affiliations:** a State Key Laboratory of Chemical Biology and Drug Discovery, Department of Applied Biology and Chemical Technology, The Hong Kong Polytechnic University, Hung Hom, Kowloon, Hong Kong SAR 999077, China; b Department of Food Science and Nutrition, The Hong Kong Polytechnic University, Hung Hom, Kowloon, Hong Kong SAR 99907, China; c College of Veterinary Medicine, Yangzhou University, Yangzhou, Jiangsu 225012, China

*Salmonella* is a notorious foodborne pathogen that comprises strains that exhibit varied ability to cause human infection. To date, this pathogen still causes over one million cases of foodborne infections annually in the United States alone.^1^ The systemic infections of high-virulence *Salmonella* strains are often seen in the nosocomial environment. The increased prevalence of antimicrobial-resistant genes in highly virulent *Salmonella* causes their invasive infection more difficult to treat. Therefore, understanding the mechanism of *Salmonella* virulence is important for solving public health issues. According to the information from the US CDC, *Salmonella* enteritidis (*S*. enteritidis) infection is the most common cause of *Salmonella* infection in clinical cases among different serotypes.^1^ We collected a set of high- and low-virulence *S*. enteritidis isolates and subjected them to comparative genomic, transcriptomic, and phenotypic analyses. The tested strains exhibited almost identical genetic composition, but over-expression of genes involved in various physiological functions was observed in the high-virulence strains. Importantly, these genes include those responsible for maltose transport, citrate metabolism, VitB12 biosynthesis, propanediol utilization, nitrite reduction, and hydrogen production. The gene knockout experiment confirmed that the deletion of these genes resulted in decreased invasiveness, reduced survival inside macrophages, reduced invasion of different organs, and lower mortality in animal experiments.

We first determined the virulence level of 61 *S*. enteritidis strains recovered from food and clinical samples using RAW264.7 cell invasion and survival assay (Table S1). It was discovered that the virulence of these isolates varied in a wide range, with internalization rate ranging from 0.0057 to 15.3199 (Fig. 1A, C) and intracellular survival rate ranging from 0.0013 to 75.5618 (Fig. 1B, D). The genetic environment of the isolates was analyzed by S1 PFGE and PCR virulotyping. The results showed that the 61 isolates all conferred 5 virulence factors including *invA*, *avrA*, *ssaQ*, *sopE1*, and *bcfC* genes, and none of them was detected to carry the Peyer's patch-specific virulence factor, *gipA* (Table S2). The S1 PFGE result also indicated that there was no significant diversity of genetic profile between the strains (Fig. S1). In total 12 isolates with diverse virulence phenotypic characteristics were further subjected to whole genome sequencing analysis (Table S3–5). The whole genome sequencing alignment result confirmed that their genetic compositions were identical, which implied that the diversity of virulence phenotype was not contributed by the genetic composition of the strain.

**Figure 1:**
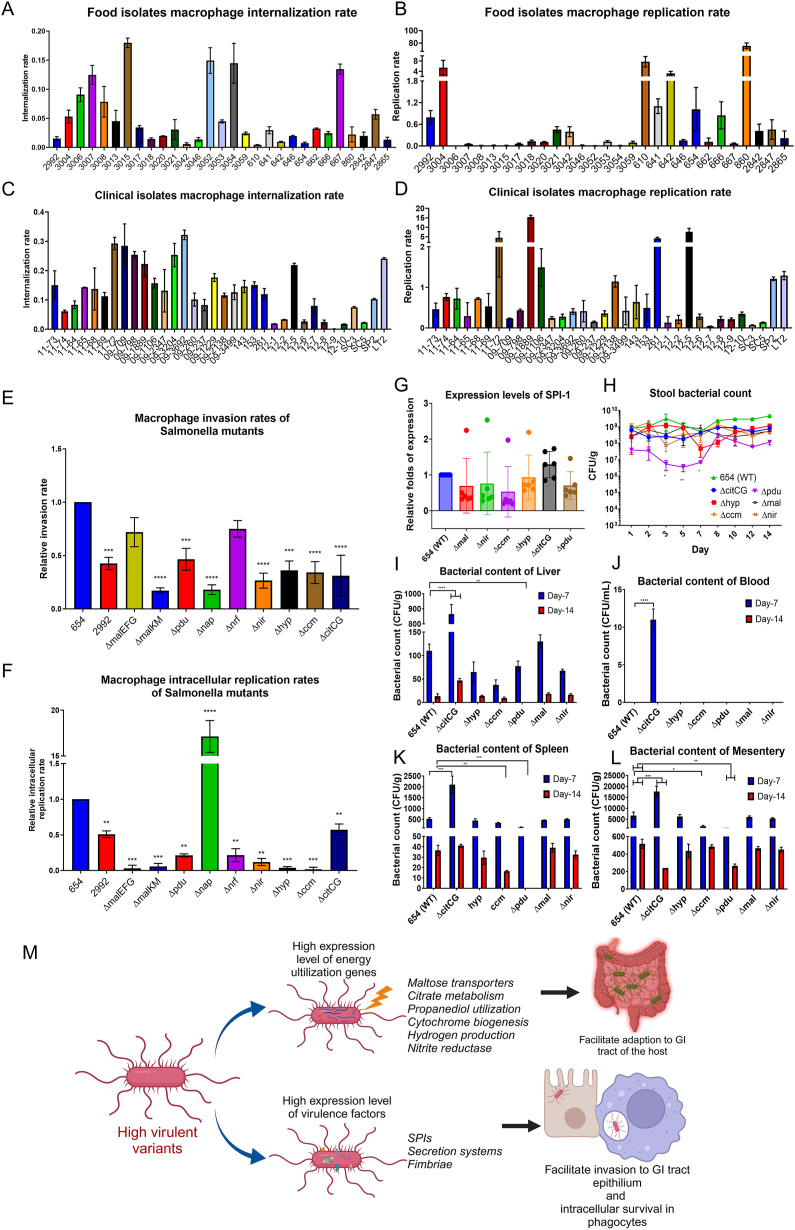
Macrophage internalization and replication rate of different *S*. enteritidis isolates collected from food and clinical specimens. **(A)** Macrophage internalization rate of food isolates. **(B)** Macrophage replication rate of food isolates. **(C)** Macrophage internalization rate of clinical isolates. **(D)** Macrophage replication rate of clinical isolates. *S*. typhimurium LT2 was used as a high-virulence reference strain. **(E, F)** Macrophage gentamicin protection assay of *Salmonella* knockout mutants. Macrophage invasion rates **(E)** and macrophage intracellular replication rates **(F)** of *Salmonella* mutants. The macrophage invasion and intracellular replication rate of the mutants were determined by gentamicin protection assay in macrophage RAW264.7. Strains 654 and 2992 were used as high and low virulence reference strains, respectively. **(G–L)** Average expression level of SPI-1 genes and *in vivo* infection assay of knockout mutants. **(G)** The expression level of SPI-1 of strain 654 and the corresponding gene knockout mutants Δ*citCG*, Δ*hyp*, Δ*ccm* Δ*pdu*, Δ*mal*, and Δ*nir* were determined using quantitative reverse transcription PCR. **(H–L)** The degree of virulence of strain 654 and the corresponding gene knockout mutants Δ*citCG*, Δ*hyp*, Δ*ccm* Δ*pdu*, Δ*mal*, Δ*nir* were determined using a mouse infection model. An equal amount of vegetative bacterial cells of each strain was introduced into the mice by the oral-feed method. Bacterial load in **(H)** stool samples was recorded for 14 days. The bacterial content of **(I)** liver tissue, **(J)** blood, **(K)** spleen tissue, and **(L)** intestinal mesentery tissue collected on day 7 and day 14 of the experiment was determined. **(M)** The flow chart illustrating the putative virulence regulatory mechanisms of *Salmonella*.

To delineate the cellular basis of differential virulence levels observed in genetically identical strains, 6 selected *S.* enteritidis strains, which exhibited different virulence levels, were subjected to RNA sequencing and then transcriptome analysis. Based on their macrophage internalization and replication rate, strains 2992 and 3046 were regarded as low virulence organisms, and 654, SE 12–5, SE 11–72, and SE 09–1889 were high virulence strains (Table S6). Gene expression analysis showed that the low virulence strains produced a lower level of mRNA in a wide range of known virulence genes, including the fimbriae synthesis pathway, virulence factors located in *Salmonella* pathogenicity island (SPI) 1, SPI2, and the secretion systems. In addition, various metabolic gene clusters including maltose transport, citrate metabolism, vitamin B12 biosynthesis, propanediol utilization, cytochrome biogenesis, hydrogen production, and nitrite reduction were highly expressed in those high virulence strains (Table S7).

The putative virulence-regulatory role of these metabolic gene clusters was tested with gene knockout experiments, followed by quantitative reverse transcription PCR analysis. The results showed that deletion of the genes encoding the maltose transporter proteins (*mal*), B12 biosynthesis and propanediol utilization proteins (*pdu*), nitrite reductase (*nir*), and cytochrome-c biogenesis protein (*ccm*) resulted in the lower expression level of the SPI-1 genes (Fig. 1G). This observation indicates that the *mal* and *nir* genes have virulence-regulatory functions and play a role in mediating the transcription of SPI-1.

To test the role of metabolic gene clusters in the expression of virulence phenotypes in *S.* enteritidis, the high virulence strain 654 was used as a model organism in gene deletion experiments to investigate the effect of gene knockout in the expression of virulence. Deletion of genes encoding the maltose transporter proteins (*mal*), cytochrome-c biogenesis protein (*ccm*), VitB12 biosynthesis and propanediol utilization proteins (*pdu*), citrate lyase (*citCG*), nitrite reductase (*nir*), and hydrogen production-related proteins (*hyp*), was found to result in a reduction in virulence level in *Salmonella* according to the macrophage invasion and survival assay (Fig. 1E, F). Furthermore, the gastrointestinal tract colonization results showed that the deletion mutant Δ*pdu* exhibited a significant reduction in the fecal bacterial count upon inoculating bacteria (Fig. 1H). The CFU recorded was over 2-log_10_ less than that of the parental strain 654 from the 3rd day until the 7th day of the experiment. Apart from the fecal samples, blood (Fig. 1J), liver (Fig. 1I), spleen (Fig. 1K), and intestinal mesentery (Fig. 1L) tissue samples were collected on the 7th and 14th day of the experiment. The result showed that the deletion of *pdu* and *ccm* genes reduced the bacterial load in the spleen, and the deletion of *pdu* and *citCG* genes reduced the bacterial load in mesentery on the 14th day (Fig. 1L), suggesting that these pathways of *S*. enteritidis were important for gastrointestinal tract colonization and invasion into the extraintestinal sites. This observation further confirms that these metabolic related pathways are important determinants of virulence in *S*. enteritidis.

In the past, the diversity of virulence phenotypes among non-typhoidal *Salmonella* strains was believed to be due to serovar variation and carriage of various chromosomal and plasmid-borne virulence genes.^2^ From the data of this study, we discovered that various metabolism pathways in *S.* enteritidis including maltose transportation, citrate metabolism, B12 biosynthesis, propanediol utilization, nitrite reduction, and hydrogen production are important for the expression of virulence phenotype in *S.* enteritidis. These metabolic pathways control different vital functions in the bacteria. Some of them are essential for energy harvesting by utilizing organic compounds or redox control of ions.^3^^,^^4^ We believe that the survival fitness of the *Salmonella* in the host could indirectly determine the virulence level (Fig. 1M). In addition, the citrate catalytic activity of *Salmonella* may affect the expression of inflammatory complexes in the host by controlling the citrate level of the intestine.^5^ The suppressed inflammatory response may help the *Salmonella* to survive under the immunity.

In conclusion, a wide range of genes that encode metabolic functions play an important role in mediating the expression of virulence phenotypes in *Salmonella*. Further studies are required to investigate mechanistic details regarding the induction of key virulence factors in the high-virulence *Salmonella* strains in different environmental niches, and why such factors are less actively expressed in the low-virulence strains even though they are genetically identical.

## Ethics declaration

Mice were purchased from the Laboratory Animal Research Unit (LARU), City University of Hong Kong, China. The animal experiments were carried out in strict accordance with the recommendations in the Animal Biosafety and Animal Handling Technique of the City University of Hong Kong, China. The animal protocol was approved by the Animal Research Ethics Committee of City University of Hong Kong, China.

## Author contributions

Bill Kwan-wai Chan: conceptualization, methodology, software, validation, formal analysis, investigation, data curation, writing-original draft, writing-review & editing. Ruichao Li: methodology, software, validation, formal analysis. Edward Wai-chi Chan: conceptualization, methodology, writing-review & editing. Kwok-yin Wong: funding acquisition, resources. Sheng Chen: conceptualization, formal analysis, funding acquisition, project administration, resources, writing review & editing.

## Conflict of interests

The authors declared that they had no competing interests.

## Funding

The work was supported by the National Natural Science Fund in China under the Guangdong Major Project of Basic and Applied Basic Research grant (No. 2020B0301030005) and the Research Grant Council of the Government of Hong Kong SAR under NSFC/RGC grant (No. NSFC-RGC, *N*_PolyU521/18).

## Figures and Tables

**figs1:**