# Supplementary material for: Novel virulence-related genes that contribute to clinical infections of Salmonella enteritidis
Source: Genes Dis. 2024 Feb 1;12(1):101236. doi: 10.1016/j.gendis.2024.101236 (PMC11489143; doi:10.1016/j.gendis.2024.101236)
Supplement: Multimedia component 1 [file mmc1.docx]

**Supplementary Materials**

**Novel virulence related genes that contribute to clinical infections of *Salmonella* Enteritidis**

Bill Kwan-wai CHAN*^a,b^*, Ruichao Li*^c^*, Edward Wai-chi CHAN*^a^*, Kwok-yin Wong*^a^*, Sheng CHEN*^ab^*^*^

*^a^ State Key Laboratory of Chemical Biology and Drug Discovery, Department of Applied Biology and Chemical Technology, The Hong Kong Polytechnic University, Hung Hom, Kowloon, Hong Kong SAR 0000, China.*

*^b^ Department of Food Science and Nutrition, The Hong Kong Polytechnic University, Hung Hom, Kowloon, Hong Kong SAR 0000, China.*

*^c^ College of Veterinary Medicine, Yangzhou University, Yangzhou, Jiangsu Province 225012, P. R. China*

**Corresponding author*

*E-mail address:* [*sheng.chen@polyu.edu.hk*](mailto:sheng.chen@polyu.edu.hk) *(S. Chen)*

*Tel: +852 3400-8619*

**Material and Methods**

***Bacterial strains***

A total of 61 *Salmonella* Enteritidis strains were included in this study, 30 of which were isolated from clinical samples in Hong Kong, three were isolated from food samples in Shenzhen, one was isolated from food sample in Hong Kong, and 29 were isolated from food samples and provided by China CDC (**Table S1**). The species identity of the test strains was confirmed by using the API 20E strips and by performing PCR assay targeting the *Salmonella*-specific *invA* gene. The serotypes of all test strains were determined by Oxoid™ Salmonella Test Kit.

***DNA techniques and PCR-virulotyping***

Genomic DNA of the test isolates were extracted from overnight culture and purified by using the PureLink Genomic DNA Mini Kit (Invitrogen). The purity and quantity of the purified DNA samples were determind by using the NanoDrop^TM^ Lite Spectrophotometer (ThermoFisher Scientific) and Qubit 4 Fluorometer (ThermoFisher Scientific). The purified genomic DNA were used as template for whole genome sequencing and PCR-virulotyping. A total of 11 known virulence determinants (included *invA*) were selected for screening (**Table S2)**. In order to characterize the virulotypes of isolates, 11 pairs of primers targeting different virulence determinants were used in PCR assay according to the previous study[^15^](#_ENREF_15).

Standard PCR was carried out in 20μL scaled volume. The PCR mixture contained PCR buffer and 0.1U rTaq polymerase (Takara), 10μM of primers, 0.2 mM dNTP, 2mM MgCl_2_ and 1μL purified genomic DNA as template. The standard cycling conditions were 95℃ for 3 mins followed by 35 cycles of 95℃ for 30s, 55℃ for 30s, 72℃ for 1 min, and a final extension step at 72℃ for 5 min. PCR amplicons were analysed by a gel documentation system (Bio-Rad).

***WGS analysis of selected S. Enteritidis isolates***

The WGS data were analysed and compared to the reference strain *Salmonella* Enteritidis PT4 isolate P125109 (NCBI accession NC_011294), >41.91 fold average genome coverage data were achieved, with average read length longer than 149 bp; over 89.39% of reads were found to exhibit a high degree of sequence homology to P125109 (**Table 3**). Each *S*. Enteritidis strain was subjected to *de novo* assembly with Celera Assembler (v8.2). Each strain could be assembled into less than 155 contigs, suggesting that the sequencing data were of high quality. The genome size was 4.7Mb in average and the CG content was between 52.09% and 52.2% (**Table S4**). Initial gene prediction was performed for each strain, with a predicted gene number of about 5135 for each strain (**Table S5**). The result of whole genome sequencing showed that slight genetic difference between clinical isolates and the food isolates existed. The high virulence *S*. Enteritidis strains were genetically identical to the low virulence strains, suggesting that the virulence level of *S*. Enteritidis was not determined by the genotype.

***RNA extraction and RNA sequencing***

The overnight culture of different bacterial strains was inoculated into fresh LB broth and allowed to growth at 37℃ with shaking until optical density reached 0.5. 5mL of log-phase culture was treated with the QIAGEN RNAprotect Bacterial Reagent. The bacterial cells were harvested by centrifugation. Total RNA was extracted by the Qiagen RNeasy Bacteria Minikit, followed by DNase treatment. The total RNA was qualified by gel electrophoresis to confirm the integrity of 23s and 16s rRNA. The ribosomal RNA in each sample was then isolated and removed using the Invitrogen^TM^ RiboMinus™ Transcriptome Isolation Kit, bacteria. The purified RNA was subjected to RNA-seq in BGI Hong Kong Co Ltd.

***Macrophage invasion and survival assay***

The virulence level of *Salmonella* strains was characterized by infecting RAW 264.7 cells and determining the internalization and replication rate. Single bacterial colonies were inoculated in LB broth and incubated at 37℃ with shaking overnight. The culture was then re-inoculated in fresh LB broth and incubated at 37℃ with shaking until the optical density of the bacterial cultures reached 0.5. The bacterial cells were harvested by centrifugation and washed once with phosphate buffered saline (PBS). The washed bacterial cells were then inoculated into PBS and the optical density was adjusted to 1.0. The bacterial suspension was added, at a rate of 1%, to DMEM cell culture medium, followed by addition to RAW 264.7 (ATCC® TIB­71™) cells pre-coated in 24-well cell culture plate with a multiplicity of infection (MOI) ratio of 10:1. The plates were then centrifuged at 500 rpm for 5 min to synchronize the infection, followed by incubation at 37C, 5% CO_2_ for 25 mins. The plates were then washed twice with pre-warmed PBS and incubated with a medium containing 200 μg/ml gentamicin for 1.5 h; the medium containing 10 μg/ml of gentamicin was then incubated for the rest of the experiment. The supernatant was removed at 2 and 16 h after infection, the cells were then washed twice with pre-warmed PBS and lysed with 0.2% Triton X-100. Serial dilutions of the lysates (10^-1^,10^-2^,10^-3^,10^-4^) were plated onto LB agar to enumerate the number of intracellular bacteria.

***Gene knockout experiments***

*Preparation of Competent cells*

The knockout mutants of *Salmonella* Enteritidis were generated by using the λ-red system. Electro-competent cells of *Salmonella* were first produced by the following steps: bacteria were inoculated into LB broth and incubated at 37℃ with shaking until OD_600_ reached 0.3, followed by fast-chill in ice-water for 10 minutes. The bacterial cells were pelleted by centrifugation at 6,500xg and the supernatant was discarded. The cells were then washed with 10% ice-cold glycerol three times. The cell pellet was resuspended in 10% glycerol and stored at -80℃.

The competent cells were then transformed with pKD46 plasmid which carried the λ-red system by using electroporation. The transformed cells were recovered by adding SOC medium and incubated at 30℃ with shaking for 1 hour. The recovered cells were spread onto LB agar supplemented with ampicillin (100μg/mL) and incubated at 30℃ overnight. Transformants carrying pKD46 were collected and inoculated into LB broth supplemented with ampicillin (100μg/mL) and further incubated at 30℃ with shaking. The pKD46 transformed *Salmonella* Enteritidis strains were re-inoculated into fresh LB broth supplemented with ampicillin (100μg/mL) and incubated at 30℃ with shaking until OD_600_ reached 0.3. Expression of the λ-red system was induced by 0.5% L-arabinose for 1 hour. The induced cells were made competent using the protocol described above. The competent cells were mixed with ice-chilled 10% glycerol and 100μL aliquots were prepared, flash-frozen by liquid nitrogen and stored in -80℃ for future use.

*Preparation of homologous DNA fragments*

A pair of primers that exhibited homology with 50bp at the 5’ side at the two ends of a target gene to be deleted was used to amplify the kanamycin resistance gene in plasmid pKD4. The sequence of primers used for producing mutants are listed in **Table S8**. PCR was performed by using high fidelity polymerase to ensure the integrity of the sequence in PCR products. The PCR products were purified by gel electrophoresis, followed by gel purification. The DNA fragments was dissolved in ultrapure water and stored at -20℃ until use.

*Transformation of homologous DNA fragments and selection of knockout mutants*

The competent cells prepared from the previous steps were thawed slowly on ice; 5μL of homologous DNA fragments were added to the thawed competent cells and mixed gently. Electroporation (E=18kV/cm) was performed upon addition of prewarmed SOC medium. The transformants were recovered at 37℃ with shaking for 1 hour. The recovered cells were spread onto agar plates supplemented with kanamycin (50μg/mL), followed by incubation at 37℃ overnight. The single colonies were inoculated into LB broth supplemented with kanamycin (50μg/mL) and incubated at 37℃ with shaking overnight. Deletion of the target gene was confirmed by Sanger sequencing. The mutants were kept viable by storing at -80℃ with 15% glycerol.

***RNA extraction and qRT-PCR***

The overnight culture of the test strains was first re-inoculated into fresh LB broth and allowed to grow at 37℃ with shaking until optical density reached 0.5; 1mL of log-phase culture was then treated with the QIAGEN RNAprotect Bacterial Reagent. The bacterial cells were harvested by centrifugation. Total RNA was extracted by the Qiagen RNeasy Bacteria Minikit, followed by DNase treatment. The quality and quantity of RNA was determined by using the Nanodrop spectrophotometer. One μg of total RNA was subjected to reverse transcription using Life technologies Superscript III reverse-transcriptase. Real-time RT-PCR was performed by using the Applied Biosystem Quant Studio 3 and the Life technologies SYBR Select Master mix. Primers used in qPCR are listed in Table S9. Melt curve analysis of PCR product was performed to ensure specificity. Expression levels of the test genes were normalized with housekeeping gene that encodes the DNA gyrase subunit B. The primers used for each qPCR reaction are listed in **Table S9**.

***Murine infection assay***

*Sepsis infection model*

ICR mice aged 5 weeks were used as the host for *S*. Enteritidis infection. Each experimental group consisting of 5 mice was infected by different mutants of *S*. Enteritidis. Briefly, S. Enteritidis mutants were first grown in LB broth until the optical density at 600nm reached 0.5. The bacterial cells were harvested and washed once with sterile 0.9% sodium chloride solution. The washed bacterial cells were then inoculated into 0.9% sodium chloride solution. The bacterial suspensions were injected into the mice through tail vein at the final dosage of 10^5^ bacterial cells. Water and food were given to each mouse during the experiment. The death rate of mice in each experimental group was recorded at 12-hour intervals.

*Gastrointestinal tract infection model*

ICR mice aged 5 weeks were used as the host for *S*. Enteritidis. Each experimental group consisting of 6 mice was infected by different mutants of *S*. Enteritidis. Each mouse was gavaged with 20 mg streptomycin daily for 3 consecutive days to eliminate intestinal microbes before the start of the experiment. Growth and treatment of *S*. Enteritidis mutants was the same as mentioned in sepsis infection model. The bacterial suspensions were gavaged into the stomach of the mice at the final dosage of 10^7^ bacterial cells. Normal water and food were given to each mouse during the experiment. The fecal samples were collected on the 1^st^, 2^nd^, 3^rd^, 5^th^, 7^th^, 8^th^,10^th^, 12^th^ and 14^th^ day after infection. The mice were dissected on the 7^th^ and 14^th^ day (3 mice on each day) and the bacterial load in various organs was determined by the plate-count approach. All animal experiments were approved by the Animal Research Ethics Sub-committee, City University of Hong Kong.

**Ethical Statement**

The animal use protocols were approved by the Laboratory Animal Research Unit of City University of Hong Kong. All animal received humane care in compliance with the Principles of Laboratory Animal Care as formulated by the National Society for Medical Research.

**Statistical analysis**

All data were presented as the mean ± SD. One-way ANOVA analysis of variance was used to calculate the differences between various experimental groups. A two-tailed value of P < 0.05 was regarded as statistically significant. *P ≤ 0.05, **P ≤ 0.01, ***P ≤ 0.001, ****P≤0.0001.

**Data availability**

The WGS and RNAseq data supported the findings in this study are available from the corresponding author upon reasonable request.

The data of qPCR, in vitro assays and animal experiments generated or analysed during this study are included in this published article and its supplementary materials file.

**Supplementary Tables and Figures**

**Supplementary Table S1. A list of 61 *S.* Enteritidis isolates collected from different sources and investigated in this study**

| **Isolates** | **Source** | **Location** | **Isolates** | **Source** | **Location** |
| --- | --- | --- | --- | --- | --- |
| **610** | food | China CDC | **09-798** | blood | CUHK |
| **641** | food | China CDC | **09-2138** | blood | CUHK |
| **642** | food | China CDC | **09-2347** | blood | CUHK |
| **646** | food | China CDC | **09-237** | blood | CUHK |
| **654** | food | China CDC | **09-260** | blood | CUHK |
| **662** | food | China CDC | **09-3204** | blood | CUHK |
| **666** | food | China CDC | **09-3499** | blood | CUHK |
| **667** | food | China CDC | **09-3692** | blood | CUHK |
| **860** | food | China CDC | **09-709** | blood | CUHK |
| **2842** | food | China CDC | **11-64** | stool | CUHK |
| **2847** | food | China CDC | **11-65** | stool | CUHK |
| **2865** | food | China CDC | **11-68** | stool | CUHK |
| **2992** | food | China CDC | **11-69** | stool | CUHK |
| **3006** | food | China CDC | **11-72** | stool | CUHK |
| **3007** | food | China CDC | **11-73** | stool | CUHK |
| **3008** | food | China CDC | **11-74** | stool | CUHK |
| **3013** | food | China CDC | **12-1** | stool | CUHK |
| **3015** | food | China CDC | **12-10** | stool | CUHK |
| **3017** | food | China CDC | **12-2** | stool | CUHK |
| **3018** | food | China CDC | **12-5** | stool | CUHK |
| **3020** | food | China CDC | **12-6** | stool | CUHK |
| **3021** | food | China CDC | **12-7** | stool | CUHK |
| **3042** | food | China CDC | **12-8** | stool | CUHK |
| **3046** | food | China CDC | **12-9** | stool | CUHK |
| **3052** | food | China CDC | **143** | stool | CUHK |
| **3053** | food | China CDC | **153** | stool | CUHK |
| **3054** | food | China CDC | **SC3** | food | Shenzhen |
| **3059** | food | China CDC | **SC5** | food | Shenzhen |
| **3064** | food | China CDC | **SP2** | food | Shenzhen |
| **09-1106** | blood | CUHK |  |  |  |
| **09-1229** | blood | CUHK |  |  |  |
| **09-1889** | blood | CUHK |  |  |  |

**Supplementary Table S2. Prevalence of known virulence genes in 61 *S.* Enteritidis isolates.**

| **Virulence Gene** | ***invA*** | ***avrA*** | ***ssaQ*** | ***mgtC*** | ***siiD***  ***(spi4D)*** | ***sopB*** | ***gipA*** | ***sodC1*** | ***sopE1*** | ***spvC*** | ***bcfC*** |
| --- | --- | --- | --- | --- | --- | --- | --- | --- | --- | --- | --- |
| Positive | 61 | 61 | 61 | 59 | 60 | 57 | 0 | 58 | 61 | 53 | 61 |
| Negative | 0 | 0 | 0 | 2 | 1 | 4 | 61 | 3 | 0 | 8 | 0 |
| Prevalence rate (%) | 100.00 | 100.00 | 100.00 | 96.72 | 98.36 | 93.44% | 0.00 | 95.08 | 100.00 | 86.89 | 100.00 |

**Supplementary Table S3. Alignment of whole genome sequences of the test *S.* Enteritidis strains to strain P125109.**

| **Sample** | **Source** | **Location** | **Number** | **Read Length** | **Base (Mbp)** | **Sequence Depth** | **Aligned %** |
| --- | --- | --- | --- | --- | --- | --- | --- |
| 642 | food | China CDC | 876,267 | 158:159 | 278.93 | 59.53 | 93.47 |
| 654 | food | China CDC | 679,058 | 156:157 | 213.81 | 45.63 | 93.8 |
| 3018 | food | China CDC | 636,439 | 162:163 | 207.92 | 44.37 | 92.67 |
| 3042 | food | China CDC | 729,614 | 159:160 | 234.03 | 49.94 | 94.32 |
| 3046 | food | China CDC | 623,684 | 156:157 | 196.38 | 41.91 | 94.8 |
| SC3 | food | Shenzhen | 942,294 | 150:151 | 284.93 | 60.8 | 90.01 |
| 09-1106 | blood | CUHK | 1,560,539 | 149:150 | 469.08 | 100.11 | 93.81 |
| 09-1889 | blood | CUHK | 1,050,085 | 162:163 | 342.68 | 73.13 | 91.39 |
| 09-260 | blood | CUHK | 945,355 | 158:159 | 300.3 | 64.08 | 89.39 |
| 09-3692 | blood | CUHK | 867,186 | 163:164 | 283.81 | 60.57 | 90.61 |
| 11-72 | stool | CUHK | 1,125,032 | 165:166 | 374 | 79.81 | 89.86 |
| 12-5 | stool | CUHK | 624,037 | 161:162 | 201.78 | 43.06 | 93.32 |

**Supplementary Table S4. *de novo* assembly profile of the test *S.* Enteritidis strains**

| **Sample** | **Contigs #** | **Total Len (bp)** | **N50 Len (bp)** | **MAX Len (bp)** | **GC Content (%)** |
| --- | --- | --- | --- | --- | --- |
| 642 | 93 | 4,696,584 | 95,744 | 382,062 | 52.14 |
| 654 | 107 | 4,680,501 | 97,631 | 257,318 | 52.19 |
| 3018 | 150 | 4,699,326 | 62,951 | 205,405 | 52.2 |
| 3042 | 84 | 4,699,722 | 102,757 | 316,125 | 52.15 |
| 3046 | 100 | 4,641,566 | 82,532 | 196,887 | 52.15 |
| SC3 | 78 | 4,709,633 | 101,288 | 524,063 | 52.14 |
| 09-1106 | 111 | 4,703,998 | 107,869 | 356,693 | 52.15 |
| 09-1889 | 124 | 4,772,272 | 73,438 | 278,408 | 52.09 |
| 09-260 | 152 | 4,712,789 | 52,815 | 295,867 | 52.15 |
| 09-3692 | 155 | 4,717,918 | 49,318 | 163,913 | 52.17 |
| 11-72 | 90 | 4,651,420 | 98,334 | 270,062 | 52.12 |
| 12-5 | 113 | 4,718,699 | 74,617 | 263,019 | 52.14 |

**Supplementary Table S5. Initial gene prediction profiles of selected *S.* Enteritidis strains**

| **Sample** | **Gene #** | **Total Length (bp)** | **Average Length(bp)** | **GC Content (%)** |
| --- | --- | --- | --- | --- |
| 642 | 5137 | 4,115,223 | 801.09 | 53.15 |
| 654 | 5061 | 4,082,892 | 806.74 | 53.22 |
| 3018 | 5094 | 4,096,074 | 804.1 | 53.23 |
| 3042 | 5128 | 4,117,962 | 803.03 | 53.13 |
| 3046 | 5087 | 4,076,754 | 801.41 | 53.08 |
| SC3 | 5181 | 4,134,972 | 798.1 | 53.08 |
| 09-1106 | 5112 | 4,104,963 | 803.01 | 53.18 |
| 09-1889 | 5238 | 4,164,000 | 794.96 | 53.08 |
| 09-260 | 5179 | 4,105,026 | 792.63 | 53.11 |
| 09-3692 | 5181 | 4,107,105 | 792.72 | 53.11 |
| 11-72 | 5094 | 4,085,550 | 802.03 | 53.08 |
| 12-5 | 5129 | 4,124,319 | 804.12 | 53.19 |

**Supplementary Table S6. The macrophage internalization and replication rate of selected *S.* Enteritidis strains which were subjected to RNA sequencing**

| **Strains** | **Source** | **Location** | **Year** | **Internalization rate** | **Replication rate** |
| --- | --- | --- | --- | --- | --- |
| 2992 | food | China CDC | 2012 | 0.0152 | 0.7934 |
| 3046 | food | China CDC | 2012 | 0.0135 | 0.0370 |
| 654 | food | China CDC | 2012 | 0.0075 | 1.0150 |
| 12-5 | stool | CUHK | 2012 | 0.2189 | 7.6038 |
| 11-72 | stool | CUHK | 2011 | 0.2925 | 4.4744 |
| 09-1889 | blood | CUHK | 2009 | 0.2228 | 15.3199 |

**Supplementary Table S7. Metabolic pathways and regulatory elements involved in mediating virulence expression in *S.* Enteritidis**

| **Fold of change*** | **Genes** | **Order in Chromosome** | **Gene function** |
| --- | --- | --- | --- |
| Regulatory gene | | | |
| 3.6 | - | SEN4082 | hypothetical protein |
| 10.2 | *gerE* | SEN4085 | GerE family regulatory protein |
| 10.2 | *araC* | SEN4086 | AraC family regulatory protein |
| 3.0 | *-* | SEN4087 | hypothetical protein |
| 5.9 | *ramA* | SEN0551 | transcriptional activator RamA |
| 7.0 | *yncC* | SEN1467 | DNA-binding transcriptional regulator |
| 6.3 | *hilA* | SEN2718 | invasion protein regulator |
| 4.7 | *yaiV* | SEN0357 | DNA-binding transcriptional regulator |
| 4.6 | *sprB* | SEN2708 | AraC family transcriptional regulator |
| 4.5 | *invF* | SEN2740 | AraC family regulatory protein |
| 3.8 | *stpA* | SEN2643 | DNA binding protein |
| 3.3 | *hilD* | SEN2717 | AraC family transcriptional regulator |
| 3.1 | *yhjB* | SEN3429 | LuxR family transcriptional regulator |
| 3.1 | *hilC* | SEN2709 | AraC family transcriptional regulator |
| 2.4 | *tdcA* | SEN3086 | DNA-binding transcriptional activator TdcA |
| 2.3 | *-* | SEN0709 | LysR family transcriptional regulator |
| 2.5 | *recO* | SEN2559 | DNA repair protein RecO |
| 2.2 | *era* | SEN2560 | GTP-binding protein Era |
| 2.2 | *rnc* | SEN2561 | Ribonuclease III |
| Fimbriae synthesis pathway | | | |
| 32.9 | *fimA* | SEN0524 | type-1 fimbrial protein subunit a |
| 29.6 | *fimI* | SEN0525 | major pilin protein |
| 45.1 | *fimC* | SEN0526 | fimbrial chaperone protein |
| 25.7 | *fimD* | SEN0527 | outer membrane usher protein FimD |
| 17.3 | *fimH* | SEN0528 | FimH protein |
| 13.7 | *fimF* | SEN0529 | fimbrial protein |
| 21.9 | *fimZ* | SEN0530 | transcriptional regulator FimZ |
| 4.0 | *fimY* | SEN0531 | fimbriae Y protein |
| 30.1 | *-* | SEN0532 | hypothetical protein |
| 6.7 | *fimW* | SEN0533 | fimbriae w protein |
| Salmonella Pathogenicity Island 1 | | | |
| 4.3 | *avrA* | SEN2707 | pathogenicity island membrane protein |
| 4.6 | *sprB* | SEN2708 | AraC family transcriptional regulator |
| 3.1 | *hilC* | SEN2709 | AraC family transcriptional regulator |
| 4.2 | *-* | SEN2710 | hypothetical protein |
| 4.4 | *orgA* | SEN2711 | cell invasion protein |
| 5.2 | *-* | SEN2712 | cell invasion protein |
| 4.6 | *prgK* | SEN2713 | pathogenicity 1 island effector protein |
| 4.4 | *prgJ* | SEN2714 | pathogenicity 1 island effector protein |
| 4.1 | *prgI* | SEN2715 | pathogenicity 1 island effector protein |
| 4.3 | *prgH* | SEN2716 | pathogenicity 1 island effector protein |
| 3.3 | *hilD* | SEN2717 | AraC family transcriptional regulator |
| 6.3 | *hilA* | SEN2718 | invasion protein regulator |
| 5.7 | *iagB* | SEN2719 | cell invasion protein |
| 5.3 | *sptP* | SEN2720 | tyrosine phosphatase |
| 4.4 | *sicP* | SEN2721 | chaperone |
| 4.3 | *iacP* | SEN2722 | acyl carrier protein |
| 4.8 | *sipA* | SEN2723 | pathogenicity island 1 effector protein |
| 4.3 | *sipD* | SEN2724 | pathogenicity island 1 effector protein |
| 3.8 | *sipC* | SEN2725 | pathogenicity island 1 effector protein |
| 4.4 | *sipB* | SEN2726 | pathogenicity island 1 effector protein |
| 4.0 | *sicA* | SEN2727 | hypothetical protein |
| 9.2 | *spaS* | SEN2728 | surface presentation of antigens protein SpaS |
| 5.9 | *spaR* | SEN2729 | secretory protein |
| 5.3 | *spaQ* | SEN2730 | secretory protein |
| 6.5 | *spaP* | SEN2731 | surface presentation of antigens protein SpaP |
| 5.6 | *spaO* | SEN2732 | surface presentation of antigens protein SpaO |
| 5.9 | *invJ* | SEN2733 | surface presentation of antigens protein |
| 6.0 | *invI* | SEN2734 | secretory protein |
| 5.4 | *invC* | SEN2735 | ATP synthase SpaL |
| 6.7 | *invB* | SEN2736 | secretory protein |
| 5.4 | *invA* | SEN2737 | secretory protein |
| 5.3 | *invE* | SEN2738 | cell invasion protein |
| 5.2 | *invG* | SEN2739 | secretory protein |
| 4.5 | *invF* | SEN2740 | AraC family regulatory protein |
| 7.4 | *invH* | SEN2741 | cell adherance/invasion protein |
| 1.7 | *pipA* | SEN0951 | pathogenicity island protein |
| 3.6 | *pipB* | SEN0952 | pathogenicity island protein |
| 5.4 | *pipC* | SEN0954 | cell invasion protein |
| 4.7 | *sopB* | SEN0955 | cell invasion protein |
| 3.0 | *orfX* | SEN0956 | hypothetical protein |
| 2.3 | *-* | SEN0957 | hypothetical protein |
| 5.6 | *SopE* | SEN1155 | type III secretion system, secreted effector protein SopE |
| 4.3 | *-* | SEN1157 | hypothetical protein |
| 4.8 | *sopD* | SEN2784 | hypothetical protein |
| Salmonella Pathogenicity Island 2 | | | |
| 5.5 | *ssaU* | SEN1623 | secretion system apparatus protein SsaU |
| >10 | *ssaT* | SEN1624 | type III secretion protein |
| 5.2 | *ssaS* | SEN1625 | type III secretion protein |
| 10.6 | *ssaR* | SEN1626 | type III secretion system protein |
| 3.4 | *ssaQ* | SEN1627 | type III secretion system protein |
| 10.8 | *ssaP* | SEN1628 | type III secretion protein |
| 20.6 | *ssaO* | SEN1629 | type III secretion protein |
| 3.7 | *ssaN* | SEN1630 | type III secretion system ATPase |
| 5.0 | *ssaV* | SEN1631 | secretion system apparatus protein SsaV |
| 10.4 | *ssaM* | SEN1632 | pathogenicity island protein |
| 11.0 | *ssaL* | SEN1633 | secretion system protein |
| 7.2 | *ssaK* | SEN1634 | pathogenicity island protein |
| 11.9 | *-* | SEN1635 | pathogenicity island protein |
| 8.5 | *ssaJ* | SEN1636 | pathogenicity island lipoprotein |
| 7.8 | *ssaI* | SEN1637 | pathogenicity island protein |
| 7.7 | *ssaH* | SEN1638 | pathogenicity island protein |
| 4.2 | *ssaG* | SEN1639 | pathogenicity island protein |
| 1.8 | *sseG* | SEN1640 | pathogenicity island effector protein |
| 2.6 | *sseF* | SEN1641 | pathogenicity island effector protein |
| 4.7 | *sscB* | SEN1642 | pathogenicity island protein |
| 8.6 | *sseE* | SEN1643 | pathogenicity island effector protein |
| 5.5 | *sseD* | SEN1644 | pathogenicity island effector protein |
| 4.7 | *sseC* | SEN1645 | pathogenicity island effector protein |
| 4.2 | *sscA* | SEN1646 | type III secretion system chaperone protein |
| 4.4 | *sseB* | SEN1647 | pathogenicity island effector protein |
| 3.1 | *sseA* | SEN1648 | pathogenicity island protein |
| 4.0 | *ssaE* | SEN1649 | secretion system protein |
| 14.7 | *ssaD* | SEN1650 | pathogenicity island protein |
| 4.7 | *ssaC* | SEN1651 | outer membrane secretory protein |
| 16.6 | *ssaB* | SEN1652 | pathogenicity island 2 secreted effector protein |
| 3.7 | *-* | SEN2228 | exported protein |
| 1.8 | *sifA* | SEN1825 | secreted effector protein |
| 4.3 | *sifB* | SEN1454 | secreted effector protein |
| 4.8 | *sopD* | SEN2784 | hypothetical protein |
| 1.7 | *pipA* | SEN0951 | pathogenicity island protein |
| 3.6 | *pipB* | SEN0952 | pathogenicity island protein |
| 5.4 | *pipC* | SEN0954 | cell invasion protein |
| 4.7 | *sopB* | SEN0955 | cell invasion protein |
| 3.0 | *orfX* | SEN0956 | hypothetical protein |
| 2.3 | *-* | SEN0957 | hypothetical protein |
| 14.5 | *sseJ* | SEN1422 | translocated effector protein SseJ |
| >10 | *-* | SEN1423 | hypothetical protein |
| 3.2 | *-* | SEN1423A | hypothetical protein |
| 1.3 | *sspH2* | SEN2224 | secreted effector protein |
| 2.1 | *sseI* | SEN0916 | type III secreted protein |
| Type I secretion system | | | |
| 18.4 | - | SEN4026 | hypothetical protein |
| 8.8 | - | SEN4027 | integral membrane protein |
| 7.7 | - | SEN4028 | type-I secretion protein |
| 5.6 | - | SEN4029 | type-I secretion protein |
| 3.0 | - | SEN4030 | hypothetical protein |
| 9.3 | - | SEN4032 | type-1 secretion protein |
| Maltose transporters | | | |
| 4.4 | *malG* | SEN3994 | maltose transporter permease |
| 4.5 | *malF* | SEN3995 | maltose transporter membrane protein |
| 4.4 | *malE* | SEN3997 | maltose ABC transporter periplasmic protein |
| 7.0 | *malK* | SEN3998 | maltose/maltodextrin transporter ATP-binding protein |
| 4.5 | *lamB* | SEN4000 | maltoporin |
| 4.5 | *malM* | SEN4001 | maltose regulon periplasmic protein |
| Citrate metabolism | | | |
| 2.7 | - | SEN0054 | sensor kinase |
| 4.2 | - | SEN0055 | oxaloacetate decarboxylase subunit beta |
| 5.0 | - | SEN0056 | oxaloacetate decarboxylase |
| 3.3 | - | SEN0057 | oxaloacetate decarboxylase subunit gamma |
| 5.1 | - | SEN0058 | citrate-sodium symporter |
| 3.5 | *citC2* | SEN0059 | [citrate (pro-3S)-lyase] ligase |
| 3.4 | *citD2* | SEN0060 | citrate lyase subunit gamma |
| 3.1 | *citE2* | SEN0061 | citrate lyase subunit beta |
| 2.7 | *citF2* | SEN0062 | citrate lyase subunit alpha |
| 2.3 | *citX2* | SEN0063 | citx protein |
| 2.3 | *citG2* | SEN0064 | citg protein |
| B12 biosynthesis and propanediol utilization | | | |
| 3.1 | *pduA* | SEN2036 | propanediol utilization protein |
| 3.3 | *pudB* | SEN2037 | propanediol utilization protein PduB |
| 3.6 | *pduC* | SEN2038 | glycerol dehydratase large subunit |
| 3.8 | *pduD* | SEN2039 | diol dehydratase medium subunit |
| 4.4 | *pduE* | SEN2040 | diol dehydratase small subunit |
| 3.9 | *pduG* | SEN2041 | propanediol utilization protein |
| 4.5 | *pduH* | SEN2042 | propanediol utilization protein |
| 3.7 | *pduJ* | SEN2043 | propanediol utilization protein |
| 3.8 | *pduK* | SEN2044 | propanediol utilization protein |
| 4.4 | *pduL* | SEN2045 | propanediol utilization protein |
| 4.5 | *pduM* | SEN2046 | propanediol utilization protein |
| 4.4 | *pduN* | SEN2047 | propanediol utilization protein |
| 4.3 | *pduO* | SEN2048 | propanediol utilization protein |
| 4.5 | *pduP* | SEN2049 | CoA-dependent proprionaldehyde dehydrogenase |
| 3.7 | *pduQ* | SEN2050 | propanol dehydrogenase |
| 2.4 | *pduS* | SEN2051 | propanediol utilization ferredoxin |
| 1.9 | *pduT* | SEN2052 | propanediol utilization protein |
| 1.7 | *pduU* | SEN2053 | propanediol utilization protein PduU |
| 1.7 | *pduV* | SEN2054 | propanediol utilization protein PduV |
| 1.4 | *pduW* | SEN2055 | propionate kinase |
| 1.7 | *pduX* | SEN2056 | propanediol utilization protein |
| Cytochrome biogenesis | | | |
| 3.2 | *ccmH* | SEN2230 | cytochrome c-type biogenesis protein H1 |
| 3.4 | *ccmG* | SEN2231 | cytochrome c biogenesis protein CcmG |
| 3.4 | *-* | SEN2232 | cytochrome c-type biogenesis protein F1 |
| 3.4 | *ccmE* | SEN2233 | cytochrome c-type biogenesis protein CcmE |
| 5.8 | *-* | SEN2234 | heme exporter protein D2 |
| 3.2 | *-* | SEN2235 | heme exporter protein C2 |
| 2.7 | *-* | SEN2236 | heme exporter protein B |
| 3.3 | *-* | SEN2237 | cytochrome c biogenesis protein CcmA |
| 4.5 | *napC* | SEN2238 | cytochrome c-type protein NapC |
| 4.9 | *napB* | SEN2239 | citrate reductase cytochrome c-type subunit |
| 5.0 | *napH* | SEN2240 | quinol dehydrogenase membrane protein |
| 5.4 | *napG* | SEN2241 | quinol dehydrogenase periplasmic protein |
| 4.8 | *napA* | SEN2242 | nitrate reductase catalytic subunit |
| 7.5 | *napD* | SEN2243 | assembly protein for periplasmic nitrate reductase |
| 4.8 | *napF* | SEN2244 | ferredoxin-type protein |
| 1.4 | *nrfA* | SEN4047 | cytochrome c552 |
| 1.7 | *nrfB* | SEN4048 | cytochrome c nitrite reductase pentaheme subunit |
| 1.7 | *nrfC* | SEN4049 | cytochrome c-type biogenesis protein |
| 2.0 | *nrfD* | SEN4050 | cytochrome c-type biogenesis protein |
| 2.9 | *-* | SEN4051 | heme lyase subunit NrfE |
| 4.2 | *-* | SEN4052 | formate-dependent nitrite reductase complex subunit NrfF |
| 2.4 | *nrfG* | SEN4053 | formate-dependent nitrite reductase complex subunit NrfG |
| Hydrogen production | | | |
| 1.6 | *hypF* | SEN2683 | hydrogenase maturation protein |
| 7.4 | *hydN* | SEN2684 | electron transport protein HydN |
| 1.0 | *hycI* | SEN2687 | hydrogenase 3 maturation protease |
| 1.2 | *hycH* | SEN2688 | formate hydrogenlyase maturation protein |
| 1.4 | *hycG* | SEN2689 | formate hydrogenlyase subunit 7 |
| 1.2 | *hycF* | SEN2690 | formate hydrogenlyase complex iron-sulfur subunit |
| 1.8 | *hycE* | SEN2691 | formate hydrogenlyase subunit 5 |
| 3.4 | *hycD* | SEN2692 | formate hydrogenlyase subunit 4 |
| 4.5 | *hycC* | SEN2693 | formate hydrogenlyase subunit 3 |
| 7.3 | *hycB* | SEN2694 | formate hydrogenlyase subunit 2 |
| 6.8 | *hycA* | SEN2695 | formate hydrogenlyase regulatory protein HycA |
| Nitrite reductase | | | |
| 6.0 | *nirB* | SEN3301 | nitrite reductase large subunit |
| 5.1 | *nirD* | SEN3302 | nitrite reductase small subunit |
| 3.4 | *nirC* | SEN3303 | nitrite transporter NirC |

**Supplementary Table S8. List of primers used for constructing knockout mutants**

| Gene | Forward Primer (5’ to 3’) | Reverse Primer (5’ to 3’) |
| --- | --- | --- |
| *pdu* | ATGCAACAAGAAGCACTAGGAATGGTAGAAACCAAAGGCTTAACCGCAGCATGGGAATTAGCCATGGTCC | TCACTGCAGTTTGACCCCGCCTGTGACCATCTTGAGTAAATGTTGTTTTGGTGTAGGCTGGAGCTGCTTC |
| *citCG* | ATGCAGAATAGAGTGAATCTAATATTTAAACGCATTTATTTGCAAAAGGAATGGGAATTAGCCATGGTCC | TTACTCAGGATAGAGGGAACCGGCAGGAAAATGACTTAAAAACCAGGTCAGTGTAGGCTGGAGCTGCTTC |
| *nrf* | ATGGCAAGGAAAACACTACGCGCACGCCGTTTCTTCAGCCTTATATTTCCATGGGAATTAGCCATGGTCC | TTATTTCTGCCGATTTTGTAACAATTTAGTCAAATTAATGGCCTCTACCAGTGTAGGCTGGAGCTGCTTC |
| *nap* | ATGGTTGATTTATCCCGTCGAAGCATGTTGACCGGCAGTTGGCGCAACGCATGGGAATTAGCCATGGTCC | TCAGAAACCTGGTTTAACATCACGCATATCCGGCAGTTTATGCGCGATCCGTGTAGGCTGGAGCTGCTTC |
| *ccm* | ATGACAATGATGCTTGAAGCCAGAGATCTGTACTGCGAGCGGGACGAGAGATGGGAATTAGCCATGGTCC | TTATTTCTCCTGCGCCAGCCGGATACTGCGCTCTATCACCGCCCGCCGGGGTGTAGGCTGGAGCTGCTTC |
| *hyp* | ATGACTATTTGGGAAATAAGCGAAAAGGCCGATTACATCGCGCAACGGCAATGGGAATTAGCCATGGTCC | CTACACTTCCCTTAACGCTCGCGCTGCGGCAATCACGCCCTGTCCAAACGGTGTAGGCTGGAGCTGCTTC |
| *malEFG* | ATGAAGATTAAAACTGGCGTAGGCATCCTCGCATTATCCGCACTTACGACATGGGAATTAGCCATGGTCC | TTAACCTTTCACACCCCCAGCCGTCAGGCCGTTGACCAGCCAACGTTGCGGTGTAGGCTGGAGCTGCTTC |
| *malKM* | ATGGCGAGCGTACAGCTACGAAATGTAACGAAAGCCTGGGGTGACGTGGTATGGGAATTAGCCATGGTCC | TTACCCCTTGCCTTTTACACTGCTGATAAAGGTGGAACGGGCAGATGTCGGTGTAGGCTGGAGCTGCTTC |
| *nir* | ATGAGCAAAGTCAGACTCGCTATTATCGGTAATGGTATGGTCGGCCACCGATGGGAATTAGCCATGGTCC | TTAATGGTTGGCTGTAACCTTAATTTGGTTGGTTTTTTGCGGAACAGGACGTGTAGGCTGGAGCTGCTTC |

**Supplementary Table S9. List of primers used in qRT-PCR.**

| Gene | Forward Primer | Reverse Primer |
| --- | --- | --- |
| *gyrB* | CAGATCTACGAGCACGGCGT | AGTGAAGGTTTCGTGGCTCGG |
| *invF* | CGCACCAGTATCAGGAGACC | TCCACTAATCCTGCGCCATC |
| *sprB* | CGAGAGAAGCTCCGTCTTCCG | ACAATACCTCTTCCGGCGTCT |
| *tdcA* | GAGGTATTTCCAAAAGCGCAGGTATCC | CGATAGCAAAGTCCAGCCGCC |
| *yaiV* | GCAGGCCCCCTTTATTTTCGGTT | AACCGCTCTCCGCAATTAATTT |
| *hilD* | CGTGACGCTTGAAGAGGTCAATGG | GTGCATAGAGAGCGCCAAGTCG |
| *hilC* | CATGCGGACTTGTTGCCAGG | CTCACATTACTACAAACCCGTGGACA |
| *hilA* | CCCGCTTCTCTCTTGCAAAACAAAATTG | ATCTGCTTTGTGTCCCAGCGAAG |
| *yncC* | TGGCGGTGGTCGCCGCTG | CCACTTTCCAGCGCCTGTTGAA |
| *yhjB* | GGATGGCAGTCAAAAGTGGCTGCA | GCGCAAGGACAAAGGTTTCGGC |
| *ramA* | TATTCCAAGTGGCACCTGCAGCGC | AGCTTCCGTTCACGCACGT |
| *stpA* | GACGCGAAGAAGAAGAATTGCAGC | CTTCCGGGTTAATACCGTCTGCTT |
| *rnc* | AAAAAGCGGCGGATTCCGTCG | GGATTAACTGCTCGACGGTCTGG |
| *invC* | AACGGAGGCGGATGTCTTTGT | AGCGCGCAACATATCCACGAA |
| *sopE* | GGCGCTATGTCGATTCCTTTGC | CAGCCAGACCCGTGAAGCTAT |
| *bcfC* | CGAGAAGGGCGGCTGAAGTAT | TTGTGTTCCGCCATACAGCGT |
| *invA* | GGCGAGCAGCCGCTTAGTATT | CCGGCTCTTCGGCACAAGTAA |
| *ssaB* | GTGATGGCCCAGCCATGGAG | GGCATCCAGCATCAAGGCATG |
| *sscA* | GCGCAGCGCGACTTTTTCAAT | TTTGAGCCTGGCAGCATTCCC |
| *ssaV* | AGCCCGACGTTACATTCTGCC | GAGGGACGCCGGTATCCTCAA |
| *ssaU* | GTACTTTTCGGGCGCTACCGT | GCCATCACCCCAACCCATAACC |
| *ssaQ* | ATGGCCAACGGGTTTTTTGCG | CGTAAGCTGGCACCAGCCTAA |
| *sopB* | ACGCTTTTTTGCGGGATACGT | GGCTTTGTTTTCAGCGCCGAC |
| *siiA* | ACGAAAGTAATCCGTGGCCT | TGGCTCCGCTATTTTGAGCA |
| *siiD* | ACCGGAGGAACAATTCAGCG | GCTTTGTCCACGCCTTTCAT |
| *siiE* | TATTGCCCCTGTACCTCCGA | GTAGACTTTGGCTCTGCCGT |
| *fimA* | GGTGACGCTGGGTCAATACCG | GCCACTTTCGGATCGCAGTCA |
| *fimC* | CAGCGAGCCCAAAAGCGAAAA | GACCGACGGGATGGCTTTCAC |
| *fimD* | TCCTACCTCTTCCAGCGGCGA | CTCACGCTGCAATAGCGGAAC |
| *fimZ* | GAATTACCGGGCACCGACGG | CCGCTCTTATTGCTCTTCCGGC |
| *malK* | CGGAGCCGCGTGTGTTTTTG | GCGGCCCAGACGTTTATGCA |
| *malG* | CCAGATGTTTCCGGCGGTTTT | GCCGCCATGCGTATTCAGACC |
| *citF2* | GCAGGCGGCTAATGTGATCGA | TTCCAGGAAACGGGTGACCGC |
| *citC2* | ATCGGCGCCATCGTCATGAAC | AGATGCAACCAGTCACAGGCC |
| *pduA* | TTAACCGCAGCCATAGAGGCCG | AGCCCGGAGCCAATCTTTTCAT |
| *pduD* | TTTTGCGCGAAGTCATTGCCGG | TTACCTTCCACGGCGACGAACG |
| *pduP* | GCGACCCAGCAGATGATGGC | CCGCTCTTCATGCCCATTGC |
| *napB* | GGAAGGGGCGATTCGTATGCC | TCGACGCTGTGCGGGATCAT |
| *ccmH* | ACGGCAACTTCGTCACCTACG | GGCAACGATTATCCACCCGCC |
| *nrfA* | GCGGGACGATTTGATCAGCAATC | ATCCCACGGGAACTTAACGGC |
| *hycA* | CGCGTTGGCGGATGGTTTTAAC | GAGCTGCGCCTGTGCAATCAA |
| *hycF* | TTCTGCGGTCGCTGCGAAGAA | AAGCGGGACTGTTGCAGGAAG |
| *hydN* | TCGCCAGTGTGAAGACGCGC | GCAGCGTTCCTGCATCACAT |
| *nirB* | TGAACGCCATCGAACTGCCG | CGGCAGTTTATCCACGCCGA |
| *nirC* | ATTTGACCGTGGCTGATGGT | GTCGGTCTCGGCATCATTCT |

**Supplementary Figure S1. Cluster analysis of PFGE profiles of 61 *S.* Enteritidis isolates.** Strains labelled as blood CUHK and stool CUHK were isolated from blood or stool samples from patients in Hong Kong. Strains labelled as food SZ were isolated from food samples in Shenzhen, while strains labelled as CDC were isolated from various food samples provided by China CDC.
